# On the Mind

**Published:** 1855-10-01

**Authors:** M. J. Rae

**Affiliations:** Physician to the Carlisle Dispensary.


### ON THE MIND.

BY M. J. RAE, M.D., L.R.C.S.E.

*Physician to the Carlisle Dispensary.*

The three great foundations of human knowledge are, nature, mind, and God. The mind, next to God, is the most important subject for the contemplation of man; and a right appreciation of its powers, nature, and destiny, is essential to his welfare and happiness, and to his elevation to a higher state of intellectual and moral existence. The human mind has been a fruitful source of daring speculation, impious assertion, and ignorant research among mankind in every age of the world. The object of this paper is, not to give a review of the various opinions which have been held by philosophers regarding the nature, the powers, and susceptibilities of the mind, nor even to enumerate its powers and functions, but to show that the mind does exist as an immaterial spiritual essence. That which thinks, feels, and reasons, we term mind: that which has extension, weight, colour, &c., we term matter. Mind and its phenomena form the internal world of thought and feeling: matter, in its varied forms and combinations, forms the external and visible world. Some philosophers have denied the existence of mind; others have disbelieved in the existence of matter; while some, again, have confounded together matter and mind as one and the same substance. Before examining the sceptical and other opinions relative to mind, we shall notice a few of the opinions that have prevailed among philosophers respecting the seat of the thinking principle in man. Aristotle located the mind, or soul, in the left ventricle of the heart. This opinion has come down even to the present time, as is evidenced by the popular phrases—a hard, or tender heart, &c. Other ancient philosophers maintained that the soul was not fixed in any particular part of the organism, but was diffused throughout the body. This opinion was revived by Whytt, a celebrated modern physiologist. The movements observed by him in the limbs of recently decapitated animals, seemed to favour the idea of the soul not being located exclusively in the brain. The function of the spinal cord was not known in Whytt's time, hence the mistake which he and others fell into with regard to the seat of the mind. There have not been wanting philosophers, who in their anxiety to find a fitting habitation for the mind, have plunged it into the stomach: and if one may judge by the very delicate, and almost exclusive attention which this organ receives from many in our own day, some vestiges of this opinion would appear to linger still in the popular mind. When we attend to our mind's thinking, we have an obscure feeling that our thoughts take place in the brain; accordingly, some ancient, and most modern philosophers, have considered this viscus to be the seat of the mind. Plato recognised three faculties of the mind; the first he placed in the liver; the second, or irrational soul, he located in the heart; and the third, or rational soul, he placed in the brain. Similar notions to these of Plato's were entertained by Galen. Some philosophical writers have restricted the seat of the mind to particular parts of the brain: thus, Descartes located the soul in the pineal gland; others in parts surrounding that gland, or in the larger cavities of the brain. Again, some philosophers have considered the mind as diffused through the brain, while others have placed the different faculties of the mind in separate portions of that organ. The Arabians, who, previous to the revival of letters in Europe, had made great progress in various branches of learning, distributed the mental functions among the ventricles of the brain. The two anterior ventricles they made the seats of sensation and imagination; the third ventricle was the seat of the understanding, and the fourth, of memory. Similar views of the location of the mental faculties were held by the celebrated Duns Scotus and Thomas Aquinas, and by some modern physiologists, as Vieussens Meyers. The most distinguished physiologists of the present day consider the cerebrum to be the seat of the intellectual powers, and the base of the brain that of the volitions and emotions. It is not yet determined what part of the brain is the seat of the mind, nor even how much of that organ is necessary to thought; in fact, notwithstanding all the efforts of ancient, modern, and living physiologists, we are yet ignorant of the function of various parts of the brain, and it may be safely affirmed, that it will require the "researches of the acutest intellects for ages to come to determine the function of the various parts of the soft, pulpy mass forming the human brain;" and therefore, it seems an unphilosophical and preposterous attempt of phrenologists to determine mental powers by an external examination of the head, when the function and uses of the parts contained within it have not been ascertained. Although the part of the encephalon, which is the seat of the mind, has not been determined, there is no doubt that it is the organ of the mind. This is evident from the fact, that without a brain no phenomena, strictly mental, are ever exhibited. It is also evident from the connexion observed between mental operations and the development of this viscus. In infancy, when the brain is very soft, and only partially developed, there is hardly any manifestation of mental phenomena. In youth, the brain attains a higher development, and brings with it a corresponding increase of mental power. In the prime of life, when the brain has reached its full maturity of growth and vigour, we recognise the mind exercising its most marvellous characteristics. From this to old age, there is, in general, a gradual declension of mental vigour, until the person "falls into the sear and yellow leaf,"—when that vigour of thought, that ready utterance, which formerly characterized him, are no more. The "voluptuous swell" of music, the empassioned eloquence and glowing imagery that charmed, now fall unheeded on his ear; the breathing statue and the fair forms of nature have now no beauty for his weakened eye. The present makes no impression on his mind,—he lives upon the past, his thoughts are of his earlier years,—on "words that run molten still in memory's mould, and will not cool." "He sinks to second childishness, sans teeth, sans eyes, sans taste, sans everything," when death steps in and closes the scene.

That the brain is the organ of mind, is further evident from the mental manifestations, varying, not only according to age, but also according to the varying condition of the brain throughout the whole period of individual existence. The disturbance of thought and feeling, the dislocations of memory, &c., in the various affections of the brain, as in fevers, inflammation, injuries, &c., all prove that the brain is the organ of the mind; that it is the seat of consciousness, the centre of sensation, the instrument by which the mind holds converse with the external world; that it is the material tenement of the mind, "the dome of thought, the palace of the soul."

We have thus represented the brain as the organ of the mind; but the sceptic denies that the thinking principle, termed mind, exists. He says it cannot be seen nor distinguished by any of the other senses, and therefore, according to his superior wisdom, it cannot exist. The sceptic must and does believe in the existence of many things which cannot be recognised by the senses, as the principle of life in plants and animals for instance. Yet he says, Show me the mind, make it visible to my sight, give me the same evidence of its existence as what is afforded of the existence of material objects, and I shall then believe it as a reality. Now, what evidence has he of the existence of matter? He talks about touching and seeing matter, but how does he know that that which he touches or sees really exists? He says, because he is conscious that he touches or sees it, and therefore is convinced of its existence. Consciousness then determines the existence of matter, without which his senses would be of no use whatever in learning either the existence of matter or one single property of it. He thinks, feels, reasons, or rather argues; he cannot touch or see these, but he is conscious of their operation within himself. If then he infers the existence of matter because he is conscious of touching or seeing it, he must also believe in the existence of mind, because he is conscious of its operation within himself. Nay, he should be even more convinced of the existence of mind than matter, because the evidence of its existence, viz., processes of thought, volitions, feelings, &c., makes a direct and instantaneous appeal to his consciousness; whereas the proofs of the existence of matter appeal to his consciousness indirectly, through his senses, which may deceive him. In the case of mind, there is no possibility of deception; because the proofs of its existence pass through no channel or mediums, but directly and at once to his consciousness. The sceptic, however, may say, that although he is conscious of thinking, feeling, and willing, that this does not necessarily imply the existence of a permanent substance termed mind. We answer, that extension, weight, form, colour, which are revealed to his consciousness by his senses, do not prove the existence of an abiding substance termed matter. If he disregards the facts of consciousness relative to mind, to be consistent, he must also disregard the facts of consciousness respecting matter, and consequently have to deny the existence both of matter and mind.

The sceptic is noisy in argument and clamorous for demonstration, forgetting all the time that there is a consciousness within man which convinces him of the existence of mind with a force and with a power such as argument never gave nor demonstration effected. Materialists admit that the brain is the organ of the mind, but, in general, deny that the mind is an essence distinct from the brain. They say that mind is merely the result of a peculiar organization of the brain; that all mental manifestations are only the result or product of material changes in that organ; that religion, thought, joy, &c., are all material, not spiritual, and that at death the mind ceases to exist. This is the common doctrine of the materialist. We would ask, how mere material changes in the brain could alone produce thought, or the glowing forms of fancy and the sublime creations of the imagination? What mere vibrations of, or changes in, organic fibres could alone produce a mother's love, a Christian's faith, a patriot's heroism; or give rise to the brilliant coruscations of genius, the fiery zeal of the enthusiast, or the divine composure of the martyr at the stake, or form the "hope that springs eternal in the human breast?" What peculiar combination of material particles alone could form the power which enables man to grasp the past and the present, to unravel the secrets of nature, to range from world to world and from system to system, to measure the distances of the stars, and calculate the comet's distant flight; that power which enables him to rise from the contemplation of the wonders of the world below, and of the mighty orbs which glitter in the firmament above, to the contemplation of Him who is eternal? What particular arrangement of organic particles alone could produce that universal belief among mankind of the existence of a supreme being—a belief common to every age of the world—common to the learned and unlearned, to the man whose mind has been expanded by science and philosophy, and to the "rude untutored Indian, who sees God in clouds and hears him in the wind?" What combination of mere material particles alone could produce the notion of self-existence, of personality; or that untiring and constantly abiding principle, the will which reigns in every man, regulates the other faculties of the mind, and enables him to rise superior to the force of circumstances?

But let us encounter the materialist on his own ground. He says the mind is nothing distinct from the brain, that intellection is mere cerebration, that thought and all mental states are the result or product of material changes in the brain, as movements of its fibres, or changes in its particles. Now, it may be asked, what puts these fibres of the brain in motion? what produces this supposed state or condition of its substance, which results in thought, in the exercise of the imagination, and other states of mind? These supposed movements could not take place without the action of a force or power equal to the effect. No change of state of material particles can occur without the operation of a force or power. This is a universal fact in nature. When we look abroad upon nature, we observe a variety of forces in continual operation. We cannot analyse them, or explain their nature. The laws which regulate them—their phenomena, are all that we know concerning them. But we can only conceive of them as essences or energies—types as it were of our own mental energy when producing bodily movements.

Thus, when two material bodies are brought within a certain distance of each other, the one attracts the other. This movement is said to take place by the law of gravitation; but gravitation is not a mere abstraction. It must be a force, otherwise it could not move a particle, nor whirl a world through space. When a magnet is suspended above a needle, this is drawn towards it. The movement in the needle is determined by the magnetic force existing in the magnet. Two chemical compounds being brought into contact with each other—two new compounds are formed. The change of state, or movement in the particles of each body, is the result of the action of the chemical force or affinity. If a seed be planted in the earth, a change in its particles soon follows. This is owing to the vital force inherent in the seed. We see, then, that no change of state or condition, or movement of the particles of a body, can occur without the operation of a force or power. Therefore, we must conclude that the movements, or changes of state or condition of the molecules of the brain, which are alleged by materialists to be the sole cause of mental states, cannot occur without the operation of a force or power; and that must be the mental force or energy. There must be, even according to the materialist's own theory of mind, something in the brain, distinct and different from itself, to produce thought; and that something must be a constantly abiding and ever-acting energy; and nothing but an immaterial spiritual essence can explain mental phenomena, especially the action of the will and the notion of self.

The powers of thought are wearied by close and continued exertion, and require rest for further and difficult efforts; and fancy has oft to rest on her airy pinions before she can take another or a wider sweep through her rich and varied domains; and the imagination, even in the most gifted genius, cannot always rise to a lofty conception; the deepest sorrow and the most tearless grief may subside from the memory, and the strongest passions ebb and flow; and because these and other powers and susceptibilities of the mind are wearied by exertion, weakened by time, and cannot always be aroused into a state of activity, shallow pretenders to philosophy think that this, which is merely an evidence of the imperfection of the bodily instrument of the mind,—that this is a proof of the material and perishable nature of the soul.

But the will is not open to this senseless objection of the materialists. No; though the powers of thought and reason may fag and fail, and fancy and imagination be often cradled in repose,—though passions and emotions may pass from the memory as a dream, yet the untiring, the ever-acting, the constantly-abiding, the indomitable will remains an unanswerable proof of the soul's divine origin,—a proof that it is something distinct from the brain—spiritual, immaterial, destined to immortality. Again:—physiology teaches us that our bodily structure is constantly undergoing a change, that its old particles are continually being removed, and that new ones supplied from the blood come to occupy their place. The brain is subject to this general law, so that a certain time it will be made up of entirely different particles from what composed it at a particular previous period of life. Although the encephalon is constantly undergoing a change, and, in the course of a long life, must necessarily be frequently wholly renewed in its molecular or nervous structure, yet amid all these changes of the brain, the individual still retains the consciousness that he is himself the same being whose infant steps were tended by a mother's care, and who now, after the vicissitudes of a long life, totters on the verge of the grave. Though he has been raised from pining poverty to boundless wealth, from mean obscurity to princely rank; though his star has risen in the palace of a king and set in a pauper's bed, or in a felon's grave; and though he has entertained, by turns, the most opposite opinions in politics, philosophy, and religion, yet through all these changes in his mental and external condition he still retains the consciousness of his personality. This could not be the case were the emotions, hopes, joys, thoughts, and actions which make up the sum of human life, merely the result or product of material changes in the brain; because if this were so, then all consciousness, all remembrance of these would cease when the particles, which are their supposed representatives, have been removed from the brain by the action of the organic law just alluded to; unless we have recourse to the absurd supposition, as mentioned by Dr. Abercrombie, that each particle, on its removal from the brain, impressed its successor with all the thoughts, ideas, &c., with which it stood itself connected. Were the emotions and other mental states nothing more than the mere result or product of cerebral conditions, the past of our lives would be a blank; the impressions and hopes of our earlier years be forgotten; life a dream, and old age a miserable, joyless existence. But the individual, from youth to age—through all the vicissitudes of a long and weary life—retains the consciousness of his personality, and remembers the joys of childhood, the dreams of youth, the struggles and hopes of his riper years, even more vividly than he does the anxieties of his old age, or the impressions of yesterday,—a proof that the mind is something different from the brain—something more permanent than time, and lasting as eternity. Another proof of the spirituality of the mind—and of its being something distinct and different from the brain, and depending on organization merely for its manifestation, and not, like the vital principle, for its existence—is that the mental powers do not, as materialists assert, decline invariably *pari passu* with the vital principle. The mind may expand and increase in vigour long after the declension of the vital principle, and may remain apparently unimpaired throughout a long life, up to the very moment of dissolution, as in the case of Franklin, Watt, Wellington, and many others. The last ten years of Franklin's long life were amongst the most important of his useful existence. Wellington was a good example of the vigour of the mind continuing with the ruins of its bodily frame. His physical vigour gradually declined until he became weak and feeble as a child; but his mind remained powerful and serene to the last. His great spirit only fled when the feeble spark which bound it to humanity was quenched in night.

The opinion, therefore, openly expressed by the French infidels of last century, and still maintained by many materialists of the present day, that the mind is nothing distinct from the brain; that all mental states are only the result of material changes in that organ—this opinion, which degrades man to a mere thinking machine, and makes him the very weather-cock of circumstances—this opinion is not only opposed to revelation, but to common sense and the laws of nature. They who have entertained this degraded opinion of the mind, have entirely overlooked the necessity of having an energy or force to set the fibres, particles, or molecules of the brain in motion, or to produce the material changes in that organ which they have supposed to be the mental states themselves. Whatever share cerebral states may have in the production of mental phenomena, it is quite evident to any one who calmly reflects upon his own mental condition, that there must be, beyond and above all cerebral states or changes, an ever-acting and abiding principle within him—an essence or energy to produce the ever-varying phases of human consciousness. The term materialism, however, must be understood with great limitations, as it has been too often applied indiscriminately to the philosophical systems of all writers, who have not fully recognised the spiritualist's theory of mind. The refined and philosophical materialism of Priestly, for instance, who did not admit the mind to be an essence distinct from the brain, although he recognised both the immortality of man and the existence of a supreme Being, has been classed with the gross materialism of the philosophers just alluded to, who not only ignored the immortality of man, but denied the existence of a Deity. Priestly's views on the nature of the soul have been greatly misrepresented, and he had to endure much undeserved and cruel persecution from the narrow-minded and fanatic of his own day and country, on account of his philosophical opinions. Even Hartley has been ranked among materialists, because he merely tried to account for the manifestations of mind by his theory of "Vibrations," although he was careful to show that the mind, the thinking principle itself, was a spiritual, immaterial essence. The more philosophical materialists of the present day recognise the mind as an essence either distinct from the brain, but material and imperishable in its nature; or, like Priestly, admit the immortality of the mind, but do not consider it to be an essence distinct from the brain. The opinion of the soul being material does not necessarily imply, as it has been foolishly supposed, a disbelief in its immortality, nor in the being of a God. Nay, some distinguished writers of the present day consider, that such a belief respecting the nature of the soul does not necessarily lead to immorality—an opinion, however, to which we cannot fully subscribe. It requires considerable powers of mind to grasp the idea of the mind being a material spirituality as it were; and the opinion is much more likely, in most minds at least, to lead to pantheism, or to an elevation of nature above God, than to an elevated idea of him. Hence the danger of its operation upon the mind and conduct of man. We have used the term material spirituality, with reference to the essence of mind, because we can only conceive of forces or powers, essentially material, as being also spiritual, as the vital and magnetic forces, for instance. The materialistic doctrine under consideration recognises the immortality of the mind, as well as the existence of a Deity. It supposes the mind to be an essence similar to the essences or forces which produce the phenomena in nature. To say that the mind is a material, and not an immaterial essence, is merely arguing about a term. We do not know, nor will we ever know, in this state of existence, the nature of the essence of either matter or mind. We cannot tell in what they differ, nor in what they agree. They are too subtle for our limited capacities to analyse. They lie beyond the grasp of the human intellect. We know only their phenomena—the results of the operation of these essences; and judging by the results of the operation of the principles, or essences, which we term matter and mind, it is unphilosophical and contrary to common sense to suppose that essences, producing results or phenomena so different and dissimilar to those of each other, can be of one and the same nature. To admit this, is to admit what is absurd and contradictory, viz., that like causes produce different and opposite effects.

What but an insane or strangely distorted mind can believe that a thought or an emotion is produced by an essence of the same nature as what forms a stone or a clod of the valley; or believe that the essence is the same, in kind, that produces the efforts of reason, the bright forms of fancy, and the visions of the imagination, as that which forms the gems of earth and of ocean, and the varied objects of the vegetable world; or believe that the essence is the same which links man to man in the bonds of brotherhood and love, as that which binds together the particles that compose the eternal hills; or believe that the essence which vibrates through humanity, through the whole family of mankind, and centres in the great Spirit of the universe, thus forming one grand, vast, and glorious spiritual community, is the same in its nature as that which maintains the ocean on its bed, the planets in their spheres, and causes them to revolve in ceaseless harmony. Further:—matter, in all its forms, can be recognised by the senses. It has extension, weight, colour. It is divisible. But is a thought divisible? Can you weigh the conceptions of the imagination, or see the goddess Reason seated on her throne? Are passions and emotions made known to us by hardness, or softness, or by any other properties by which material objects manifest themselves to our senses? Matter, then, is made known to our consciousness through the senses—mind is recognised by consciousness alone. Is it not reasonable, therefore, to conclude, that essences producing results or phenomena so very different and dissimilar to those of each other, and which are revealed to us through different channels, are themselves dissimilar in their nature? Are we not justified in believing, that wonderful as are those hidden principles in nature—those essences or forces which are in continual operation in the earth, air, and ocean, forming and supporting the wonders of the outward and material creation, that they are different in kind and nature from that spiritual essence—the divinæ particula auræ which forms the internal world of thought and feeling in man. The immortality of the soul is denied by infidels and the majority of materialists, not by all of the latter, as we have just seen; for materialism is not necessarily associated either with infidelity or disbelief in the immortality of the soul. Strange that men, who have been professed worshippers of nature, should ever have conceived that the principle of thought within them would sink into nothing, or that the stability of nature did not lead them to form more rational views regarding the destiny of the soul. Nature still labours with undiminished power and vigour and skill in her innumerable and diversified workshops, from which she sends forth forms as perfect and as exquisitely beautiful as those which she first launched forth into the watery world, or sent teeming from the surface of the earth. Thousands of years have not been sufficient to diminish, far less exhaust, her creative powers; yet, threescore years and ten, or the appointed time of man's pilgrimage on earth, nay, even a few moments of time, have been thought sufficient to annihilate what is superior to and above all nature, the creative energy in man. But apart from revelation, it is contrary to the spirit of true philosophy and to the common feeling of humanity to suppose, with the sceptical materialist, that the soul at the death of its material tenement ceases to exist. The death of the body is merely another name for change of state or condition. None of its elements are lost. This can be proved by chemistry to be the case. Is it, then, philosophical to suppose when the material and grosser part of our nature merely undergoes a change at death, that the immaterial, the finer part, the thinking principle within us, shall cease to be? Decay in the animal and vegetable worlds is only a state of change, not an annihilation. The same holds good with respect to mere dead matter. Its disappearance from view is no evidence of its annihilation. It is merely a change in the condition or arrangement of its constituents. The rocks and cliffs that guard our coasts, by the incessant action of the air and the constant play of the surge upon them, may, in the course of ages, undergo some change in their rugged aspects; but the particles which crumble from their surface, and are washed away by the billows, are not lost. They are deposited in other, and it may be, distant parts, entering, perhaps, into the formation of other rocks, which may form the bulwarks of future islands, on which civilizations higher than our own may flourish, and which may be destined to form the strongholds of liberty in future and distant ages of the world. When the death of the body and the decay of animal and vegetable forms are merely indicative of a state of change, and the disappearance of inanimate matter is only a resolution into other compounds; when no material particles cease to exist, how absurd to suppose that that which is spiritual and immaterial shall cease to be. Judging then by the continued existence of matter, is it not right to infer the continued existence of mind? Does reason, independent of revelation and the aspirations of man, not justify the belief that the immaterial principle within us shall survive the dissolution of our bodies, "the wreck of matter and the crush of worlds?" Nay, when we see mere inanimate matter becoming possessed of new powers, or assuming more lovely forms by a change in the arrangement of its component parts, or disappearing from view merely to come forth again more ravishingly beautiful; when we see the black, opake, and almost valueless carbon, by a change in its particles, becoming the brilliant and transparent diamond which "glitters with the play of a thousand colours upon the hand of beauty;"—when we see these changes in mere dead matter, would it not be more reasonable to conclude that the soul at the death of its corporeal tenement, instead of sinking into nought, or even remaining as it was before, shall be endowed with purer and loftier aspirations, with more exquisite powers and susceptibilities to enable it to get a deeper insight into the majesty, the beneficence, and wisdom of Him who is eternal? But the proof of the indestructibility of the mind does not rest alone upon the analogy afforded by the permanency of matter, nor upon the evidence furnished by the continual existence of its material tenement, but upon those longings after immortality which are native to and inherent in the mind—those aspirations common to humanity, those feelings and emotions implanted in man, which convince him with a power, stronger than argument, that the principle of thought within him is immortal—"That the grave is not its goal. Dust thou art, to dust returnest, was not spoken of the soul."

We have seen that the opinion held by one class of materialists, that the mind is nothing distinct or different from the brain, that it is only the result of a peculiar organization of that organ, is not only opposed to revelation, but to common sense and the laws of nature.

There is, however, nothing degrading to human nature, as many theologians and spiritualist philosophers would seem to believe, in the supposition that the brain undergoes some change in the condition of the substance during mental operations. This we believe to be the case, and to be necessary to their occurrence; and that the brain may be worn out, not merely indirectly, but directly, by excessive thought and emotion, just as any other organ of the body may be exhausted by continued physical excitement. In every act of mind, from the highest intellectual effort to the most dreamy and tranquil exercise of the imaginative powers, — from the strongest burst of passion to the gentlest impulse that stir the soul, there are certain changes effected in the nerve-matter of the brain. This we may reasonably infer from the mysterious union which subsists between the spiritual and corporeal elements in man, without being liable either to the charge of materialism, or of detracting from the powers and qualities naturally belonging to an immaterial spiritual essence. In all probability, the changes which take place in the brain concurrently with the mental operations will never be ascertained. The light of science may never penetrate this obscure psychico-physiological region.

But the thoughts, emotions, and other mental states are not, as materialists suppose, the result or product of concurrent cerebral conditions. These material changes, whatever they may be in their nature, are not the thoughts and emotions themselves, neither are they the cause of them; the cause is mind, the spiritual or mental force. We know nothing, nor will we ever know anything, in this state of being, of the mind thinking separated from, and independent of, the brain. The union of the mind with the brain is necessary to mental manifestations, as heat and moisture are to the development of the vital force in a seed, or as the sun is to the present diffusion of light to the earth. Heat and moisture are conditions necessary to the springing up of a seed, but they are not the cause of it. The cause is the vital force inherent in the seed. Without the vital force, heat and moisture would be of no avail in the evolution of a seed into a plant. The brain is also the condition necessary to mental manifestations, but it is not the cause of them. Materialists continually confound the condition with the cause of mental phenomena. They recognise the organ, but not the essence of thought; the material, but not the spiritual element in the phenomena of mind. The brain is the organ as well as the instrument of the mind. The spiritual principle in man works, not merely through, but by means of the cerebrum. One great cause, we believe, of the slow progress of mental philosophy, and of its limited success in promoting either the intellectual or moral improvement of man, as well as of its comparative inefficiency in advancing the treatment of mental and other diseases, is, that it has, in general, been based upon the idea of the mind being an entirely independent spiritual essence—the connexion of the mind with the brain having been either wholly overlooked, or the encephalon having been viewed merely as the material tenement of the mind—the instrument by which the spiritual principle holds converse with the external world, and nothing more. And the failure of the systems of mental philosophy, grounded on cerebral physiology, has been owing to their having ignored the existence of the immaterial and spiritual principle altogether. This has been the case too often even with phrenology. Undoubtedly phrenology has been the means of promoting sounder views on physical and moral education, and of extending our knowledge of the physiology of the brain, as well as of establishing clearer views of its diseases and their treatment, whilst it may be admitted that the fundamental principles of phrenology are, in the main, correct—viz., that the brain is the organ of the mind, that it is a double organ, and that certain parts of it subserve particular functions of the mind. Yet, as a science, it is completely defective: its basis is sound, but the superstructure is thoroughly deficient. The details, in fact, are neither metaphysically nor physiologically correct. The mental analysis, corresponding to the phrenological system is utterly at variance with the facts revealed to any one who reflects upon his own inward consciousness. And bumpology is not only not established by physiological research, by pathology and comparative anatomy, but they actually disprove it. Before the advocates of phrenology can expect to see it established as a science, and elevated, as they desire, to a branch of metaphysics, two things are absolutely essential—viz., first, a comprehensive and complete analysis of mind; and secondly, a division of the encephalon corresponding to that analysis, and determined, not by an external examination of the head, but by physiological and pathological researches on the brain.

Mental and moral philosophy, as well as mental pathology, to be of real practical value to mankind, must be inductive, not speculative; they must be based, not on either the mind or brain exclusively, but on both; they must have a psychico-physiological foundation. The great aim of the metaphysician should be to investigate the mind in the way pointed out long ago by Lord Bacon—viz., in its relations to the brain. He should endeavour to discover the relations of mind and matter; the dependence of psychical on physical states; the correlation of mental and nerve force; the mutual actions and reactions of the spiritual and somatic elements in man, and all the varying phases of human consciousness in their outward manifestations. The metaphysician, in short, should not only closely and perseveringly scrutinize the facts of consciousness on the one hand, but he should also carefully investigate the facts of physiology on the other. In no other way, we believe, can mental philosophy be advanced and rendered subservient to the progress of man, and made available to the physician in the treatment of insanity and other diseases. It would also be the means of effectually putting down, or at all events of checking, the freethinking and materialism which so largely prevails at the present time, and which will always prevail so long as the corporeal and spiritual elements in man are not mutually recognised by philosophers in the production of mental phenomena.

The difficulties in the way of the establishment of a system of mental philosophy on a psychico-physiological foundation, are, as it has been truly observed in an editorial article in the *Psychological Journal* for July, very conceivable; but however great the difficulties may be, they must be approached and overcome before we can hope to see mental philosophy become of that great utility to man, to the psychologist, and the physician which it ought to be. And we venture to remark, that had the mind been studied in the manner mentioned by Bacon, and had even one-half of the genius, ability, and research been directed to that mode of mental investigation which has been wasted in metaphysical battles between the nominalists and realists, in useless speculations respecting the essence of mind and matter, and in the establishment of philosophical systems on exclusive and mistaken foundations, mental philosophy would not have been in the anomalous position which it now is, but would either have been wrought into a system commanding general acquiescence, or else, its fundamental principles would have been so correctly established as to render the completion of the details a matter of easy accomplishment to future philosophers.

